# One-year-later spontaneous EEG features predict visual exploratory human phenotypes

**DOI:** 10.1038/s42003-022-04294-9

**Published:** 2022-12-12

**Authors:** Miriam Celli, Ilaria Mazzonetto, Andrea Zangrossi, Alessandra Bertoldo, Giorgia Cona, Maurizio Corbetta

**Affiliations:** 1 Padova Neuroscience Center (PNC), University of Padova, Padova, Italy; 2 Department of Neuroscience, University of Padova, Padova, Italy; 3 Department of Information Engineering, University of Padova, Padova, Italy; 4 Department of General Psychology, University of Padova, Padova, Italy; 5 Venetian Institute of Molecular Medicine (VIMM), Padova, Italy

**Keywords:** Cognitive neuroscience, Visual system

## Abstract

During visual exploration, eye movements are controlled by multiple stimulus- and goal-driven factors. We recently showed that the dynamics of eye movements –how/when the eye move– during natural scenes’ free viewing were similar across individuals and identified two viewing styles: static and dynamic, characterized respectively by longer or shorter fixations. Interestingly, these styles could be revealed at rest, in the absence of any visual stimulus. This result supports a role of intrinsic activity in eye movement dynamics. Here we hypothesize that these two viewing styles correspond to different spontaneous patterns of brain activity. One year after the behavioural experiments, static and dynamic viewers were called back to the lab to record high density EEG activity during eyes open and eyes closed. Static viewers show higher cortical inhibition, slower individual alpha frequency peak, and longer memory of alpha oscillations. The opposite holds for dynamic viewers. We conclude that some properties of spontaneous activity predict exploratory eye movement dynamics during free viewing.

## Abstract (web summary)

Resting state oscillatory activity is shown to predict visual exploration phenotypes, with reported correlations between resting state and eye movement dynamics recorded one year before.

## Introduction

Neuroscience has traditionally examined the function of neurons and brain regions using an outside-in approach. Neural activity recorded during the presentation of stimuli or performance of behavioural tasks is correlated with stimulus or task features. This is based on the idea that the brain ‘learns’ during development stimuli and tasks by entraining neural activity out of random noise. However, neuron-to-behaviour correlation strictly depends on the researcher’s knowledge about the experimental paradigm. As recently discussed^1^, other neurons in the brain without knowledge of the experimental paradigm would have a hard time deciding if the recorded pattern of neural activity has indeed anything to do with the stimulus or task of interest, as compared to many other patterns simultaneously present.

An alternative approach to studying the brain is inside-out. In this framework, the brain comes with preconfigured and self-organized dynamics that constrain how it views and acts on the world. During development and individual experience, these intrinsic or endogenous patterns organize themselves to induce highly structured robust yet flexible patterns that statistically match the environment and the body. Accordingly, patterns of brain activity recorded in a data-driven manner both at rest and during tasks can be used to classify, predict, or model stimuli or behaviours. As more and more relationships are found, then it should be possible to understand how ‘intrinsic’ brain signals modulate during stimulus processing or behaviour of interest. In this paper, we employ this inside-out strategy to test whether electroencephalographic (EEG) spontaneous (resting state) activity recorded one year later from the original experiment distinguishes two types of observers (static, dynamic) during free viewing exploration of naturalistic visual images.

We constantly explore the visual world using complex sequences of eye movements that are driven by multiple factors: low-level visual features^2^, contextual information^3^, and task goals^4^. Eye movement features like latencies, accuracies, and velocities, underlie individual differences^5^, which are stable across time periods up to 2 years^5–12^. This observation is consistent with fMRI data showing robust interindividual differences in functional networks that are relatively independent of task states and day-to-day variability (e.g. ref. ^13^).

Classic models of visual exploration have emphasized the importance of salience, i.e. the relative sensory distinctiveness of objects in the environment, in guiding exploratory eye movements^2^. However, a recent study^14^ showed that various stimulus-based visual exploration models account for just a small portion of the variance in eye movement patterns (i.e., the best model reaching 34% of maximum information gain). Vision also relies on temporal strategies, and temporal neural codes to extract and represent spatial information^15^. In other words, it is not only important for ‘where’, but also for ‘how’ and ‘when’ to look.

In a recent study^16^, we measured inter-individual differences in a free-viewing eye movement exploration paradigm. Observers (*n* = 120, final sample *N* = 114) visually inspected a large number (*n* = 185) of naturalistic pictures, some containing human or man-made figures, and some outdoor/indoor visual scenes. The dynamics of eye movements (e.g., fixation duration, number, direction, and amplitude of saccade, etc.) across hundreds of observers and pictures were low dimensional and were described by three principal components accounting for ~60% of the variability. The first component (PC1) separated two kinds of viewers: ‘static’, characterized by longer fixations, and ‘dynamic’, characterized by shorter fixations. Critically this latent variable was independent of image saliency and semantics and was correlated with power law similarity of eye movements suggestive of intrinsic biological constraints^17^. Notably, the two kinds of observers could be also accurately classified from eye movements recorded in the absence of any stimulus (blank screen) (see also ref. ^18^). These findings suggested that intrinsic dynamics, rather than stimulus content, controlled ‘how/when’ people looked at images. In control analyses, we showed that the location of fixations (‘where’ to look), in contrast, depended strongly on stimulus information (saliency, semantics).

Given the dependency of eye movement dynamics on intrinsic dynamics, here we test whether evidence for this latent variable can be identified in spontaneous recordings of brain activity. To this end, we recruited from the previous experiment^16^ 43 participants who were representative of the two viewing styles and recorded high-density electroencephalographic (EEG) activity during eyes open and closed in the absence of any task one year after the original experiment. This activity can be considered intrinsic, not task-dependent since it was recorded one year after the behavioural session, and subjects were unaware of the purpose of the EEG study. While we could have recorded EEG both during task and rest, we opted for just recordings at rest to truly look at spontaneous brain activity not confounded with task activity or even instructions related to the task.

Subjects’ viewing style was operationalized based on the scores of the first principal component (PC1) (in ref. ^16^) that summarizes eye movement features stable over long periods of time^5–12^. The two groups of subjects recorded in this experiment showed extreme positive (static) or negative (dynamic) loadings on the PC1 score.

Our hypothesis is based on the theory that spontaneous activity plays a fundamental role in cognition by providing a generative predictive model of spatiotemporal patterns of activity during behavioural tasks^19^. Hence, we hypothesized that differences in eye movement dynamics were related to stable individual differences in the brain’s intrinsic EEG oscillatory activity. Spontaneous brain activity is behaviourally relevant in different species, and at different spatial and temporal scales (LFP^20^, EEG^21^, fMRI^22^). Moreover, the well-known reciprocal influence of task-related and spontaneous activity^23^, makes spontaneous activity (resting state) a good candidate for the prediction of trait-like behaviours (e.g. refs. ^21,24^).

We considered three EEG metrics shown to be good behavioural predictors: resting-state frequency power^25,26^, individual alpha-frequency^27^ and long-range temporal correlations^28^. The resting state frequency power is thought to reflect the baseline level of cortical activation (e.g. ref. ^29^). The individual alpha-frequency (IAF) has an established relationship with inhibition and speed of processing^30^ that could be related to the faster saccadic dynamics of the dynamic viewing style. Finally, long-range temporal correlations (LRTCs) measure the temporal structure of oscillations and have been related to behavioural fluctuations (e.g. ref. ^28^). Power-law form LRTCs have been suggested to represent underlying biological constraints (e.g. excitation/inhibition balance^31^), and were more representative in the behavioural experiment of the more static viewing style.

This study highlights two resting state oscillation profiles which predict different visual exploration phenotypes. These results suggest a link between resting-state brain activity and oculomotor behaviour.

## Results

### Spectral analysis

**Fig. 1:**
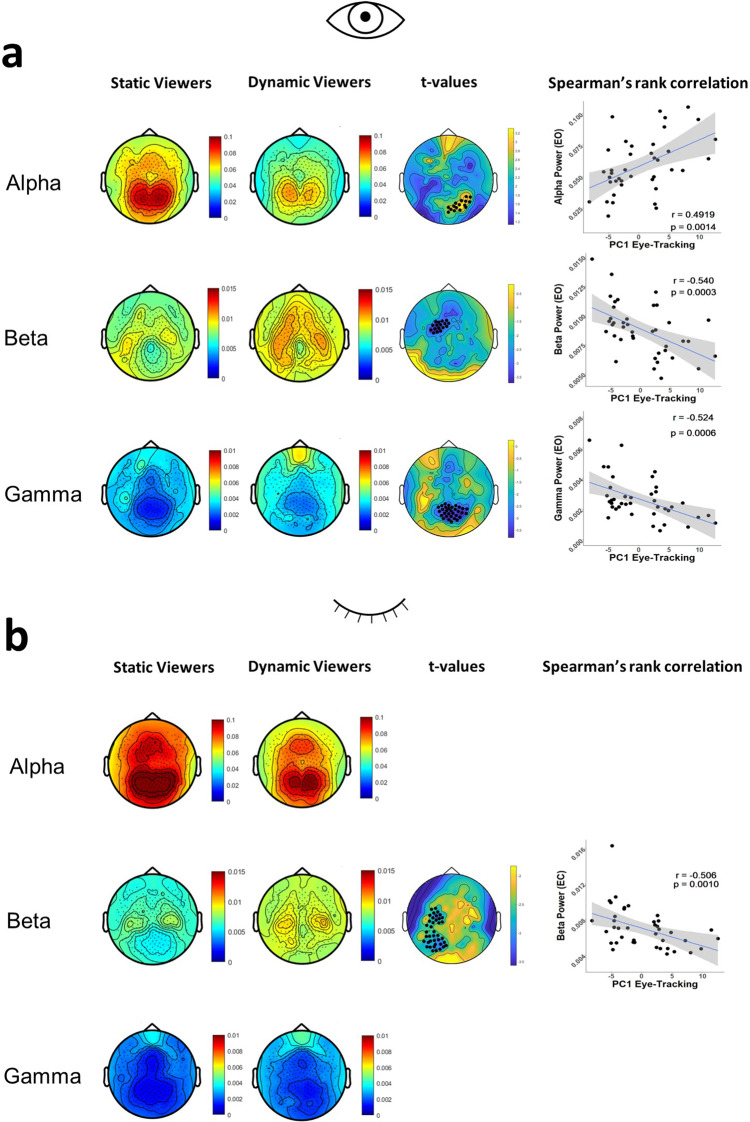
Spectral analysis results. **a** Group scalp maps in eyes open condition for alpha (7.5–12 Hz), beta (12.5–32 Hz) and gamma (32.5–45 Hz) relative power and *t*-value maps (where the comparison yielded significant results) for the cluster-based permutation analysis. Black dots index significance with cluster alpha at *p* < 0.01 (two-tailed) and alpha *p* < 0.05 (two-tailed). The right panel shows the Spearman’s rank correlation between PC1 and averaged power in the significant cluster of electrodes (with Spearman’s *r*, *p*-value and 95% CI). *N* = 40. **b** Group scalp maps in eyes closed condition for alpha (7.5–12 Hz), beta (12.5–32 Hz) and gamma (32.5–45 Hz) relative power and *t*-value maps (where the comparison yielded significant results) for the cluster-based permutation analysis. Black dots index significance with cluster alpha at *p* < 0.01 (two-tailed) and alpha *p* < 0.05 (two-tailed). The right panel shows the Spearman’s rank correlation between PC1 and averaged power in the significant cluster of electrodes (with Spearman’s *r*, *p*-value and 95% CI). *N* = 40.

In the eyes open condition, alpha, beta, and gamma bands showed significant differences between groups (ANOVA alpha band: *F*1,38 = 5.39, *p* = 0.04; beta band: *F*1,38 = 6.68, *p* = 0.04; gamma band: *F*1,38 = 4.71, *p* = 0.04; FDR corrected), thus all were subsequently compared at a scalp level with a nonparametric permutation approach with cluster correction. In the eyes closed condition, the beta band showed a significant difference between groups (*F*1,38 = 7.31, *p* = 0.03; FDR corrected) (Fig. 1).

In the eyes open condition, a significant cluster of electrodes, primarily located in the occipital regions, showed significantly higher *t*-values in the alpha band (7.5–12 Hz) in Static than in Dynamic Viewers (number of electrodes = 16, *p* = 0.01, FDR corrected). A Spearman’s rank correlation between global alpha power and PC1 values showed a positive correlation in the significant cluster (*r* = 0.4919, *p* = 0.0014, FDR corrected).

In the eyes open condition, in the beta band (12.5–32 Hz), a significant frontal cluster of electrodes showed significantly lower *t*-values in Static Viewers than in Dynamic Viewers (number of electrodes = 17, *p* = 0.01, FDR corrected). Again, we computed a Spearman’s rank correlation between beta power in the significant cluster and PC1. There was a negative correlation between global beta power and PC1 value (*r* = −0.54, *p* = 0.0012, FDR corrected).

In the eyes open condition, in the gamma band (32.5–45 Hz), a significant cluster of electrodes, primarily located in occipital electrodes, showed significantly lower *t*-values in Static Viewers than in Dynamic Viewers (number of electrodes = 32, *p* = 0.01, FDR corrected). The subsequent Spearman’s rank correlation showed a negative correlation between global gamma power and PC1 values in the significant cluster (*r* = −0.524, *p* = 0.0012, FDR corrected).

In the eyes closed condition, in the beta band (12.5–32 Hz), a significant cluster of electrodes showed significantly lower *t*-values in Static Viewers than in Dynamic Viewers (number of electrodes = 40, *p* = 0.01, FDR corrected). A Spearman’s rank correlation between beta power in the significant cluster and PC1 was significant (*r* = −0.506, *p* = 0.0013, FDR corrected).

In summary, Static viewers were characterized by stronger occipital alpha power, and weaker frontal beta and occipital gamma power (the opposite for Dynamic viewers). PC1 values were positively correlated with alpha power but negatively correlated with beta (both eyes open and closed) and gamma power.

For effect sizes see Supplementary Tables 1 and 2 and Supplementary Fig. 1.

The direction of the correlation was confirmed even when static and dynamic viewers were considered as separate groups (Supplementary Fig. 3).

### Individual alpha frequency

**Fig. 2:**
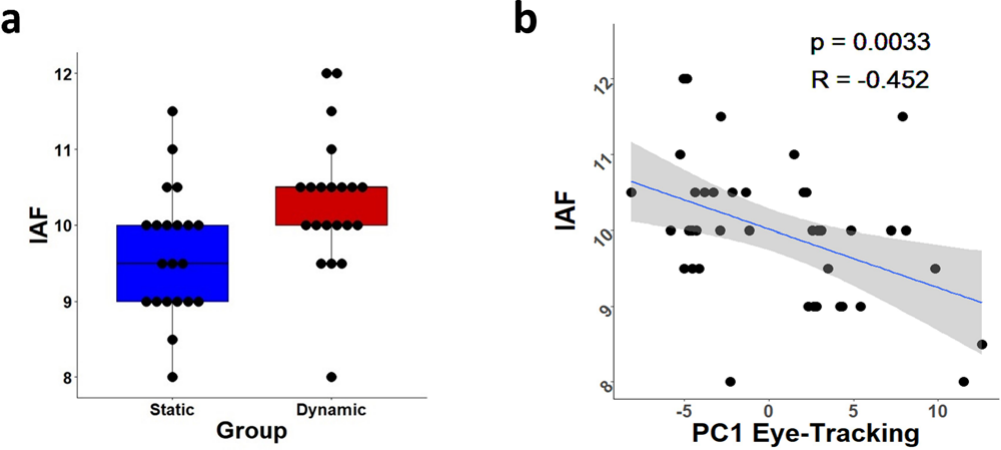
Individual alpha frequency results. **a** Individual alpha frequency values by group (static *n* = 19, median = 9.5 Hz; dynamic *n* = 21, median = 10.5 Hz). *N* = 40. **b** Spearman’s rank correlation between PC1 and Individual Alpha Frequency values (with Spearman’s *r*, *p*-value and 95% CI). *N* = 40.56.

Dynamic Viewers (i.e., subjects with shorter fixations) showed a significantly higher IAF than Static Viewers (i.e., subjects with longer fixations) (independent sample *t* = −3.324; *p* = 0.003). The Spearman’s rank correlation between IAF values and PC1 was negatively related (*r* = −0.452, *p* = 0.003) in the direction that lower PC1 scores (i.e., shorter fixations) corresponded to higher IAF (Fig. 2).

### Long-range temporal correlations

To the best of our knowledge, this measure has never been examined in eye movements. As a first step, we computed a detrended fluctuation analysis (DFA) on the eye-tracking time series to test the hypothesis that the eye movement time series showed fractal properties. To fit this hypothesis the exponents are expected to fall in the range 0.5–1. All DFA exponents extracted from the eye movement time series fell in the range 0.5–1 demonstrating long memory and LRTCs in the signal^32,33^.

The second hypothesis regarded the association between behavioural DFA exponents and PC1. These two measures partially overlap: they represent different aspects of fixation timings (i.e., PC1 is a static measure, while DFA exponents represent the temporal structure of fixations). The two measures are expected to be strongly correlated, given the fact that they represent different aspects of the same phenomenon. The two measures were significantly correlated (Spearman’s rank correlation, *r* = 0.465, *p* = 0.002).

The third step was to test the association between mean brain and behavioural exponents across subjects. There was a positive correlation between brain and behavioural exponents in the eyes open condition alpha band (Spearman’s rank correlation, *r* = 0.40, *p* = 0.009). To explore the scalp topography of the effect, we computed Spearman’s rank correlation on an electrode-to-electrode basis by using the nonparametric permutation approach with cluster correction. A significant positive cluster of electrodes was primarily located in occipital areas (number of electrodes = 30, *p* = 0.007). Next, we computed a Spearman’s rank correlation between alpha band DFA exponents in the significant cluster and behavioural DFA exponents. There was a positive correlation between alpha band DFA exponents and behavioural DFA exponents (*r* = 0.519, *p* = 0.0006). Alpha band exponents significantly differed between static and dynamic viewers (*t* = 2.03, *p* = 0.04).

**Fig. 3:**
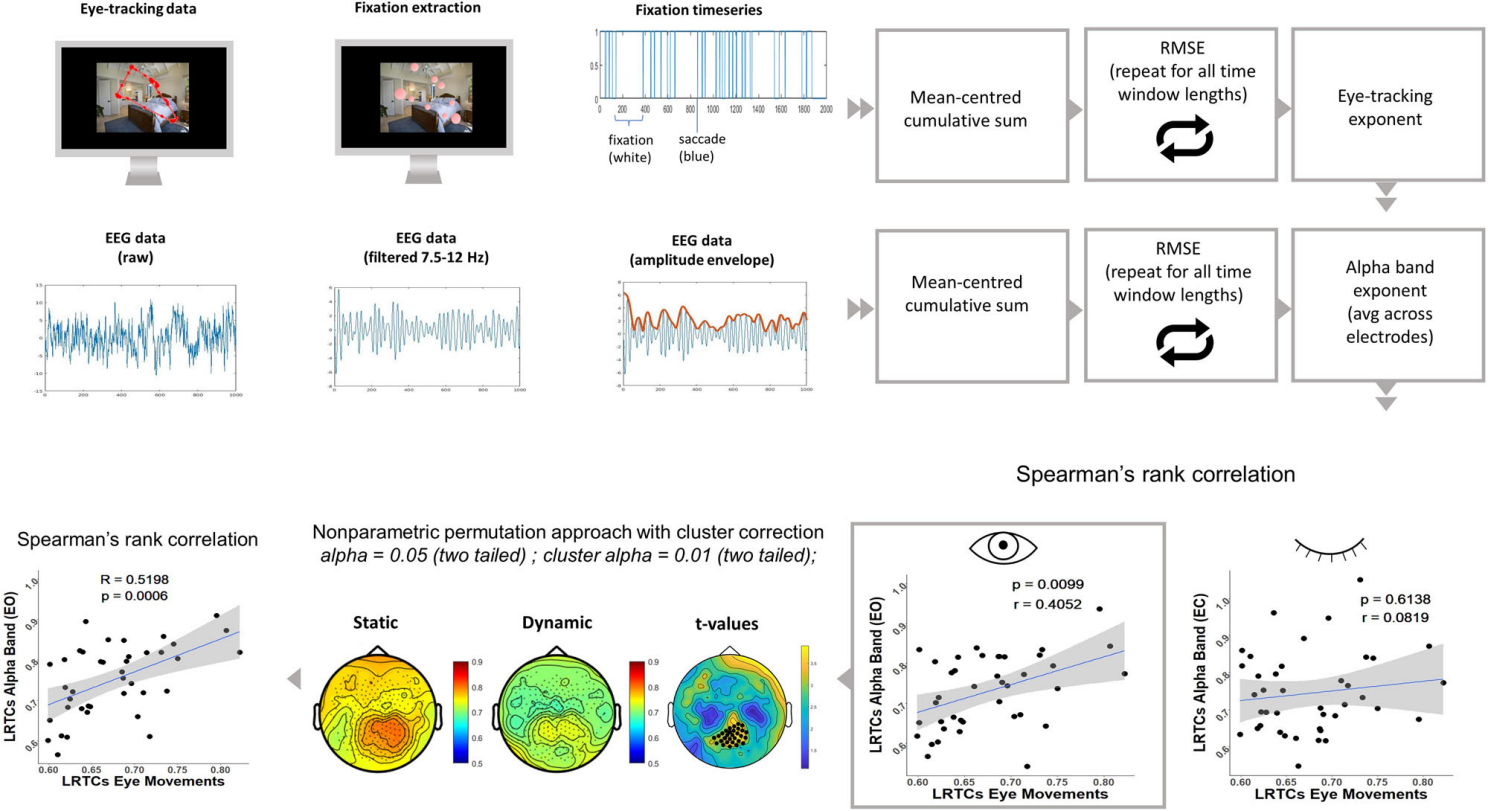
Procedure and results for DFA analysis. Workflow for DFA analysis. For eye-tracking data, after extracting fixations with a velocity-based algorithm, a fixation timeseries is built (where 0 = fixation; 1 = saccade). EEG data are the first bandpass filtered in the frequency of interest (7.5–12 Hz, filter order = 66), then the amplitude envelope is computed. For both timeseries, DFA analysis is performed. First, a correlation between eye-tracking exponents and mean alpha band exponents (i.e., averaged across 256 channels) is computed. A significant positive correlation is found in the eyes open condition (*r* = 0.405, *p* = 0.009). In this condition, Spearman’s rank correlation coefficients are computed in each electrode between alpha band DFA exponents and eye-tracking DFA exponents. Null hypothesis testing is conducted by using the nonparametric permutation approach with cluster correction. Black dots index significance with cluster alpha at *p* < 0.01 (two-tailed) and alpha *p* < 0.05 (two-tailed). Finally, a Spearman’s rank correlation is computed between DFA exponents in eye movements and DFA exponents in alpha band in the significant cluster of electrodes (*r* = 0.455, *p* = 0.003). All the scatterplots show Spearman’s *r*, *p*-value, and 95% CI. *N* = 40. LRTCs long-range temporal correlations, RMSE root-mean-square error.

The correlation between alpha band DFA exponents and behavioural DFA exponents was not present in the eyes closed condition (Spearman’s rank correlation, *r* = 0.08, *p* = 0.61) (Fig. 3).

### Control analysis on global power

To rule out the possibility that the observed differences in viewing styles were explained by differences in arousal, we contrasted global alpha power with eyes closed and the alpha reactivity index, respectively, a measure of baseline arousal and a measure of arousal reactivity (see ref. ^34^). There were no differences between the two groups of observers in either measure (Supplementary Fig. 2).

## Discussion

In a previous study^16^, we showed that eye movement exploration dynamics (how/when we move the eyes) in 120 (final sample *N* = 114) observers across 185 pictures were low dimensional. Three linear components accounted for most of the variability across pictures and subjects. The first component PC1 loaded on the duration of fixation (among other features) identifying two groups of observers: static viewers, characterized by longer fixation duration statistics and a higher similarity of gaze steps (i.e., the Euclidean distance between two consecutive gaze positions) to a power-law distribution; and, dynamic viewers characterized by shorter more frequent fixations and a lower similarity of gaze steps with a power law distribution. Critically PC1 scores were not explained by the saliency and semantic information of the objects presented in each picture. Furthermore, the two viewing styles during visual exploration were successfully classified based on the eye movement features recorded when looking at a blank screen (rest). These findings indicate that how/when we move the eyes during visual exploration partly reflects intrinsic (endogenous) dynamic mechanisms.

Based on the theory that spontaneous activity plays a fundamental role in cognition providing a generative model of spatiotemporal patterns of activity employed during behavioural tasks^19^, we tested whether these two patterns of eye movement dynamics were related to intrinsic brain dynamics, as measured by properties of EEG oscillations at rest. Accordingly, we recruited from the previous experiment^16^, 43 participants who were representative of the two viewing styles (they had opposite extreme PC1 scores) and recorded their resting state EEG one year later.

We show that visual exploration styles have robust correlates in spontaneous EEG oscillations recorded one year later. Three different oscillatory EEG features correlated with dynamic eye movement features (PC1 scores). Static viewers showed higher alpha power and lower gamma power in occipital electrodes. They also showed lower beta power in frontal electrodes. Static viewers displayed a lower individual alpha frequency. Finally, static viewers’ brain oscillations and eye movement time series were endowed with stronger long-range temporal correlation indicating a higher self-affinity and complexity.

In contrast, dynamic viewers’ oscillation profile was characterized by lower alpha power and higher high-frequency power (beta and gamma). Dynamic viewers also showed a higher individual alpha frequency and weaker long-range temporal correlations, thus a less complex signal (i.e., closer to white noise) both in brain and eye fixation time series.

Our interpretation is that these intrinsic EEG features represent a trait-like constraint on exploratory eye movement dynamic features. We propose that this constraint on eye movement dynamics depends on the timing of oscillations (i.e., IAF and LRTCs) and baseline level of cortical activation, which has been proposed to index the focus of attention (e.g. ref. ^35^). Herein we discuss data relevant to this interpretation.

Resting-state frequency power may reflect the baseline level of cortical activation (e.g. ref. ^29^). The interplay between activation (i.e., high-frequency activity, typically observed in task-relevant areas^36^) and inhibition (i.e., alpha activity, typically observedat rest^37^ or in task-irrelevant areas^36^), has been proposed to reflect the focus of attention, respectively directed externally to environment stimuli or internally to memory, emotion, cognitive information. For instance, in their seminal study, Ray and Cole^38^ found lower alpha power during external tasks, e.g. counting words in a passage, and higher power during internal tasks, e.g. mental arithmetics. Therefore, alpha rhythms may reflect a possible index of internal vs. external attention, with alpha power, decreasing linked to externally directed attention^35^. In this line of thought, then, a higher alpha power (i.e., the static viewer profile) would index a heightened inhibition with regard to external stimuli.

At the other end, dynamic viewers have lower inhibition towards external stimuli and are more focused on external stimuli. Their EEG power profile is consistent with stimulus processing^39^ and selective attention to stimuli^40^: occipital alpha power decreases—alpha desynchronization^36^—and gamma power increases^40^. This change in the balance of oscillatory power is thought to reflect the release of inhibition in sensory and task-relevant areas. According to this interpretation, dynamic viewers start from a resting profile that is more similar to that seen during stimulus processing and selective attention to stimuli^36,40–42^. While the mutual relationship between inhibition and stimulus processing is widely studied during tasks, recent studies also found a correlation between alpha and beta band resting state EEG activity and visual attention^25,26^.

In contrast to this interpretation of alpha inhibition/gamma excitation stand the results on the frontal beta band power that was lower in static than dynamic viewers, both during eyes closed and eyes open. Beta power is classically described as the inhibitory rhythm of the motor cortex (e.g. ref. ^43^), and it becomes desynchronized during movement planning^44^. This is like alpha occipital power that is higher at rest but desynchronizes during visual processing. However, a more recent interpretation is that beta rhythms represent the status quo or maintenance of a specific task or motor set^45^. This is based on evidence of increased beta synchronization during tasks in which a set is maintained over time (e.g., a working memory task). A related account is that beta rhythms in centrally connected regions of the cortex (hubs) in multiple networks maintain a prediction or prior about the temporal structure of visual exploratory behaviour^46^. This is based on the similarity of beta band power fluctuation temporal structure at rest and during visual exploration. Our results suggest that static viewers maintain at rest a more reactive motor cortex. The difference with dynamic viewers supports the notion that spontaneous activity patterns predict exploratory behaviour as in ref. ^16^.

Overall then the occipital power results indicate that static viewers have a profile of baseline cortical activation biased toward cortical inhibition and internal processing, while dynamic viewers have a profile biased toward cortical excitation and external processing. This is in line with the cognitive profiles of these two types of subjects, with static observers showing a slightly stronger visual working memory, and dynamic observers a weaker inhibition to salient but irrelevant stimuli^16^.

In addition to power relationships, we found an association between visual exploration timings (i.e., PC1 scores and fixations time-series) and timing constraints of alpha oscillations (i.e., IAF and LRTCs).

In the literature, the IAF has been correlated with speed of processing, with a weak but significant relation between IAF individual differences and reaction times^27^. A more general interpretation is that the speed of information processing could be related to the speed or frequency of the alpha pacemaker. A more recent view^30^ explains IAF in terms of neural inhibition timings. For instance, a higher IAF is linked to shorter P1 latencies^47^. This is also consistent with the relationship between higher alpha power and lower individual alpha frequency (e.g. ref. ^48^). In this view, alpha band dynamics have an active role: the inhibition serves to establish a highly selective activation pattern. Higher IAFs index stronger intra-cortical inhibition and more highly specialized activation patterns eventually resulting in faster task performance. In line with these views, dynamic viewers endowed with a higher IAF can disengage faster from fixation and generate a higher number of fixations overall. The negative relationship between IAF and PC1 scores indexing fixation duration is consistent with this interpretation. These results predict that dynamic viewers shall be faster in visual processing and attention tasks.

The final link between eye movement dynamics and brain dynamics is the observation of LRTCs in both fixation sequences and brain oscillations. Static viewers show stronger LRTCs both in the occipital alpha band and eye movement time-series. Long-range temporal correlations indicate that these signals maintain memory over time. In contrast, the fixation and oscillatory sequences of dynamic viewers were closer to white noise, i.e., they have a less temporal structure in time resembling more a random process. Power-law form LRTCs are thought to index intrinsic systems constraints (e.g., structural constraints^49^, physiological constraints^50^) that may cause recursive regularities in brain signals^51^ and behaviour^28^. The stronger link between LRTC and static viewers is highly consistent with a profile of activity and dynamics that emphasizes internal processes.

In the setting of the review, several important issues were raised that are worth discussing. One objection is whether the distinction between static and dynamic viewers can be considered stable one year later, at the time of EEG recordings. In other words, is it possible that we are documenting brain correlates of a ‘state’ recorded one year earlier not a stable ‘trait’, i.e., a true difference in viewing styles? The two groups were called back for EEG recordings based on their extreme (positive, negative) PC1 scores. PC1 scores were computed based on eye movement features that in the literature are stable over time^5,7,9,10,52^. Also in ref. ^16^, we carried out several controls showing that PC1 scores were stable in different sub-samples of even/odd images (i.e., all *r* values > 0.97); different image categories (i.e., all *r* values = 0.97). In addition, PC1 features measured at rest when viewing a blank screen classified with >80% accuracy static and dynamic viewers when looking at natural images, and vice versa. Nonetheless, we recognize that this is a relative limitation of the study.

Another point is that our findings may reflect differences in overall arousal/motivation between groups (e.g. ref. ^53^). The arousal theory of motivation states that each individual has its own baseline level of arousal, with low-arousal subjects showing less high-frequency power and more low-frequency power^54^, higher eyes closed alpha power^34^, lower alpha reactivity^55^. These features have been related to extraversion^56^ and sensation seeking^57^.

In additional control analyses, we show that static and dynamic viewers do not differ in eyes closed alpha power (*t* = 1.58, *p* = 0.12), or alpha reactivity (*t* = 0.45, *p* = 0.65; eyes closed to eyes open alpha power ratio; Supplementary Fig. 2). They also show no differences in extraversion^16^, while static viewers, those with longer fixation and higher alpha power, were more open to new experiences^16^.

Taken together these results do not suggest a difference in overall arousal/motivation. However, this experiment was not designed to test this hypothesis and future work is needed to clarify this issue.

An alternative hypothesis is that differences in brain rhythms, specifically alpha, can modulate online eye movement behaviour^58^. In other words, dynamically during a task, periods of lower alpha power immediately follow saccades, while periods of fixation are associated with increased alpha power. This would result in lower overall alpha power in those individuals who show high oculomotor activity (i.e., more saccades) during the recording. This interpretation is not inconsistent with our findings. Hebbian plasticity^59^ predicts that repeated patterns of neural activation leave a persistent trace in the brain, first in the form of synchronized spontaneous activity, later on in the form of synaptic and structural connectivity changes. Accordingly, subjects during development and/or experience might have developed a more ‘static’ or ‘dynamic’ style of eye movements during visual exploration, which has entrained in turn tonic differences in their spontaneous activity recorded in this experiment. This idea suggests that genetic differences may be partly responsible for the different developmental/experience trajectories (as in ref. ^60^).

That a complex behaviour like eye movement visual exploration can be summarized with a few dynamic features across many subjects and pictures was surprising^16^. A growing behavioural literature focused on the covariance across subjects is showing that many apparently complex behaviour underlies a low dimensionality. Apparently complicated hand movements^61^, human navigation in cities^62^, and variability in reward inhibition^63^ can be all explained by a small number of latent variables. There is also growing evidence for low dimensionality of coding through correlated neuronal activity across many neurons, as in the case of face perception^64^, hand movements^65^, and exploratory face movements^20^. Even more unexpected, somehow, is that these dynamic eye movement features are related to resting-state oscillatory properties of EEG signals recorded one year later. We think that the results suggest that the timing of oscillations (IAF, LRTCs) and inhibition (alpha power) could be key in controlling the duration of fixation and shifts thereof.

Given the high temporal resolution of both eye movement and EEG recordings, it will be interesting in future studies to explore jointly EEG signals and eye movement recordings and relate resting to task dynamics.

A final consideration is the potential of eye movement and resting-state EEG recordings as a diagnostic or prognostic tool for pathologies in which structural, metabolic, or biological measures are either too expensive or invasive. We are thinking of eye movements^66^, as well as alterations in resting state EEG metrics^67,68^ in high-impact pathologies like Alzheimer’s disease.

## Methods

### Participants

Participants for the resting-state EEG session (*n* = 43, mean age 24.11 years, SD = 2.41, 16 males) were recruited from the pool of subjects of a previous study^16^. All participants signed informed consent before the experimental session, and after it, they received a remuneration of 10€ for their participation. The study was approved by the Ethical Committee of the University of Padova.

Participants were selected based on the PC1 scores from the previous study. The PC1 is the first principal component extracted from a PCA on 59 eye-tracking features computed on a free visual exploration task on 185 pictures and 120 (final sample *N* = 114) subjects. Participants were chosen for this session based on PC1 values higher than 1 and lower than −1. These values represented the extremes of the distribution identifying subjects who belonged to the static vs. dynamic group. Eligibility in the previous study was based on the applicants being in good health, having no history of neurological disease, and having no colour blindness. All participants had normal or corrected-to-normal vision. This session took place after a mean interval of 11 months (327 days ± 43). Three participants were excluded due to the bad quality of the data, resulting in a final sample of 40 participants (19 static viewers and 21 dynamic viewers).

### Stimuli and apparatus

EEG activity was recorded with a high-density EEG system consisting of a 256-channel Hydrocel Geodesic Sensor Net, a high-impedance amplifier Net Amp 400 and Net Station Software 4.3 (Electrical Geodesics Inc.). Before testing, impedances were measured and adjusted until they were kept below 50 kΩ. Impedance of each channel was also measured and saved at the end of the session. All electrodes were referenced online to the electrode placed over the vertex (Cz in the 10/20 international system). EEG data were digitized with a sampling rate of 500 Hz. During the registration, the participant sat in a chair in a sound-shielded Faraday recording cage. A screen displaying a fixation cross was set on the centre of a desk in front of the participant. Two resting-state EEG sessions (eyes open and eyes closed) lasting 10 min each were collected. In the eyes open condition, participants were asked to look at the fixation cross for the whole duration of the task (i.e., eye movements were discouraged); in the eyes closed condition, participants were asked to keep their eyes closed for the whole duration, to relax and not to fall asleep.

Electrode positions were digitized by means of the Occipital 3D Structure Sensor (Occipital Inc.) mounted on an iPad Pro^69^ at the end of the resting-state EEG session. Structural MR T1w data (TR/TE: 7.20/3.29 ms; 165 sagittal slices; voxel size: 0.53 × 0.53 × 1.1 mm; FA: 9°; acquisition matrix: 448 × 448) were acquired in a separate session using a 3 T Ingenia Philips whole body scanner (Philips Medical Systems, Best, The Netherlands) equipped with a 32-channel head-coil, at the Neuroradiology Unit of the University Hospital of Padova, Italy. Anatomical images were obtained for 34 participants out of 43 recruited for the EEG session. Electrode positions and T1 structural images were acquired for a future study and will not be used in this work.

### EEG pre-processing

EEG data were pre-processed using Matlab (The MathWorks, Inc, Natick, MA, USA) scripts based on functions from the EEGLAB software (version 14.1.2b^70^). First, data were band-pass filtered (cut-off frequencies: 0.5–47 Hz) using two zero-phase Kaiser-windowed sinc FIR filters (high-pass: transition bandwidth = 1 Hz, order = 1812, low-pass: transition bandwidth = 2, order = 908) as suggested in Widmann et al.^71^ and resampled at the 250 Hz. Then, automated detection of the noisy channels was performed and later confirmed by visual inspection. The selection was based on the combination of the following five criteria, whose thresholds were determined with a preliminary examination of the dataset to optimize the detection: (i) impedance at the end of the acquisition above 100 kΩ; (ii) correlation to the surrounding channels less than 0.75, (iii) and (iv) standard deviation bigger than 4 for the spectral and improbability tests; (v) standard deviation bigger than 7 for the kurtosis test. Channels selected by the criterium (i), (ii) or at least two of (iii)–(v) criteria were interpolated using spherical splines^72^. Afterward, EEG data were re-referenced to the average of all electrodes. Ocular, muscular and movement artefacts were removed by applying independent component analysis (ICA) using a deflation-based fast fixed‐point ICA algorithm (http://research.ics.aalto.fi/ica/fastica/) with the hyperbolic tangent as a cost function. Independent components (ICs) were classified into seven classes (brain, muscle, eye, heart, channel noise, line noise, and other) using the ICLabel toolbox^73^. All the components classified as brain or other (but with the brain as the second highest probability) were kept. After this step, residual artefacts were corrected using the artefact subspace reconstruction (ASR)^74^. Noisy data segments were identified using a 1-s sliding-window principal component analysis (PCA). If PCs exceeded 30 standard deviations of the cleanest part of the dataset, current data was repaired using a mixing matrix computed on the cleanest portion of the data.

### Spectral analysis

The power spectral density was computed using Welch’s overlapped segment averaging estimator using spectopo() function on EEGLAB^70^ to obtain a frequency resolution of 0.5 Hz. The power spectral density was extracted for every channel and converted from dB to µV^2^/Hz. Each spectrum was then normalized to the relative frequency power by dividing the spectrum by the power computed in the whole spectrum via trapezoidal integration.

### Individual alpha frequency peak

The individual alpha frequency peak was defined as the highest absolute value in the 7–13 Hz range. The alpha peak was first automatically detected based on this criterion. All spectra were then visually checked to ensure the selected frequency was a true peak rather than the maximum value at the boundaries of the predefined alpha range. The analysis was performed on the average spectrum across occipital electrodes (cf. ref. ^75^) in an eyes-closed condition, which is the most stable measure of IAF according to the literature—i.e. it has the greater test–retest reliability^76^.

### LRTCs computation

To assess long-range temporal correlations (LRTCs), we used the detrended fluctuation analysis as described in ref. ^33^. Briefly, this technique allows to extract a scale-free exponent which describes the temporal structure of the signal in terms of self-similarity (i.e., scaling of a statistical property across scales) and long memory. This is done by assessing the rate at which fluctuations (i.e., mean-squared residuals) grow as a function of the scale (i.e., RMSE are assessed at different scales and the relation between RMSE and window length in double-log determines the DFA exponents).

Similarly to Palva^28^, here LRTCs are computed for both brain (i.e., alpha band filtered EEG) and behavioural data (i.e., fixation timeseries). Brain exponents (i.e., the temporal structure of the alpha rhythm) and eye movements exponents (i.e., the temporal structure of eye movements) are then correlated. The DFA scaling exponent is typically ranging between 0.5 and 1 in brain signals^51^ and eye movements time-series^77^. While an exponent of 0.5 index an uncorrelated signal (i.e., white noise), an exponent of 1 index has strong long-range temporal correlations^32,33^.

A more detailed version of DFA computation can be found in Supplementary Methods.

### Statistical analysis (spectral analysis)

The statistical analysis was implemented in FieldTrip toolbox^78^.

Frequency power was first averaged into five frequency bands (delta 1–3 Hz, theta 3.5–7 Hz, alpha 7.5–12 Hz, beta 12.5–32 Hz, gamma 32.5–45 Hz). Global frequency power (i.e., averaged across all 256 channels) was then compared between groups with a bin-by-bin one-way ANOVA in both conditions separately. Null hypothesis testing was conducted comparing the results of each bin-by-bin ANOVA against a null distribution of 1000 permuted datasets. All *p*-values were FDR corrected^79^. For the frequency bands showing significance between groups' differences, we conducted a comparison at the scalp level by using the nonparametric permutation approach with cluster correction^79^. This analysis was performed excluding face electrodes. We computed two-tailed independent samples *t*-tests with 1000 resamples, two-sided 95% confidence intervals, corresponding to an alpha level of 0.05 (two-tailed). Cluster threshold setting was based on maximum cluster size, with an alpha level set to 0.01 (two-tailed).

To check the extent to which these differences were linked to visual exploration styles, we also computed Spearman’s rank correlation between PC1 values and frequency power in alpha, beta, and gamma only for the electrodes identified in the previous contrast (FDR corrected). Spearman’s rank correlation was chosen due to the distribution of PC1 values after a Shapiro–Wilk Normality test suggested a non-gaussian distribution of the variable (*p* < 0.001).

### Statistical analysis (individual alpha frequency)

After testing for normality of the distribution of the IAF values (Shapiro–Wilk Normality test; *p* = 0.459; *p* = 0.051), null hypothesis testing was conducted comparing the results of the independent sample t-test against a null distribution of 1000 permuted datasets.

Subsequently, we also tested for the association between Individual Alpha Frequency and PC1 by computing Spearman’s rank correlation, to confirm that the observed group difference was linearly related to the PC1 values. Spearman’s rank correlation was chosen due to the non-Gaussianity of the PC1 distribution.

### Statistical analysis (LRTCs)

Brain exponents in the alpha band (7.5–12 Hz) were averaged across all 256 electrodes (cf. ref. ^29^). According to the literature (see for example, ref. ^28^), alpha band at rest is the band showing a more remarkable relationship with power-law-form LRTCs in behaviour.

In both eyes open and eyes closed conditions, the normality test of the distribution of the DFA exponents of the alpha band amplitude time series (Shapiro–Wilk Normality test) suggested evidence for a non-gaussian distribution (*p* < 0.001; *p* < 0.001; *p* < 0.001; *p* < 0.001). As a consequence, we computed Spearman’s rank correlation between brain and behavioural exponents.

In the eyes open condition only we computed the Spearman’s rank correlation coefficients between alpha band DFA exponents in each electrode and behavioural DFA exponents and conducted null hypothesis statistical significance testing by using the nonparametric permutation approach with cluster correction^80^. We computed 1000 resamples; two-sided 95% confidence intervals, corresponding to an alpha level of 0.05. Cluster correction method: maximum cluster size. Cluster threshold (non-parametric) was set to 0.01 (two-tailed). Finally, Spearman’s rank correlation is computed between DFA behavioural exponents and DFA alpha exponents only for the cluster of electrodes identified in the previous step.

### Control analyses

To control for possible confounding effects on PC1, we contrasted between groups all available demographic information from Zangrossi et al.^16^ and the current study (see Supplementary Table 3). After accounting for multiple comparisons, Age is the only variable significantly different between the two groups (W = 69; p corr. = 0.007). The significant difference in Age between the two groups confirms what was already seen in the previous study with the full sample^16^.

To rule out that the observed differences are not explained by differences in arousal, we contrast global eyes closed alpha power and alpha reactivity index (eyes open-eyes closed alpha power ratio^55^) between groups. The two measures are respectively a measure of baseline arousal and a measure of arousal reactivity. For further control analyses see Supplementary Information.

### Statistics and reproducibility

R (v 4.1.0) and MATLAB (r2018b) software were used to perform all analyses, the required tools and packages are cited in the Methods section (see the sections “EEG pre-processing”, “Spectral analysis”, and “Statistical analysis”). Preprocessing steps and statistical analyses are described in the “Methods” section (see the sections “EEG pre-processing” and “Statistical analysis”). All analyses are performed on the same sample (*N* = 40).

### Reporting summary

Further information on research design is available in the Nature Portfolio Reporting Summary linked to this article.

## Supplementary information

Supplementary Information

Reporting Summary

## Data Availability

The data that support the findings of this study are available from the corresponding author on reasonable request. The source data used for the main figures are available at https://osf.io/wnxkq/.
