# Supplementary material for: One-year-later spontaneous EEG features predict visual exploratory human phenotypes
Source: Commun Biol. 2022 Dec 12;5:1361. doi: 10.1038/s42003-022-04294-9 (PMC9744741; doi:10.1038/s42003-022-04294-9)
Supplement: Supplementary file 1 — Supplementary Information [file 42003_2022_4294_MOESM1_ESM.pdf]

	Cohen's d	Effect Size	CI 95% min	CI 95% max
**Global Alpha Power** Eyes Open	0.73	medium to large	0.073	1.39
**Global Beta Power** Eyes Open	-0.818	large to very large	-1.48	-0.15
**Global Gamma Power** Eyes Open	-0.68	medium to large	-1.34	-0.27
**Global Beta Power** Eyes Closed	-0.85	large to very large	-1.52	-0.18

Supplementary Table 1. Cohen's d, effect sizes and 95% confidence intervals for group comparisons in bin-by-bin global frequency ANOVA.

	Cohen's d	Effect Size	CI 95% min	CI 95% max
**Individual Alpha Frequency**	-1.05	large to very large	-1.73	-0.368

Supplementary Table 2. Cohen's d, effect sizes and 95% confidence intervals for the individual alpha frequency group comparison.

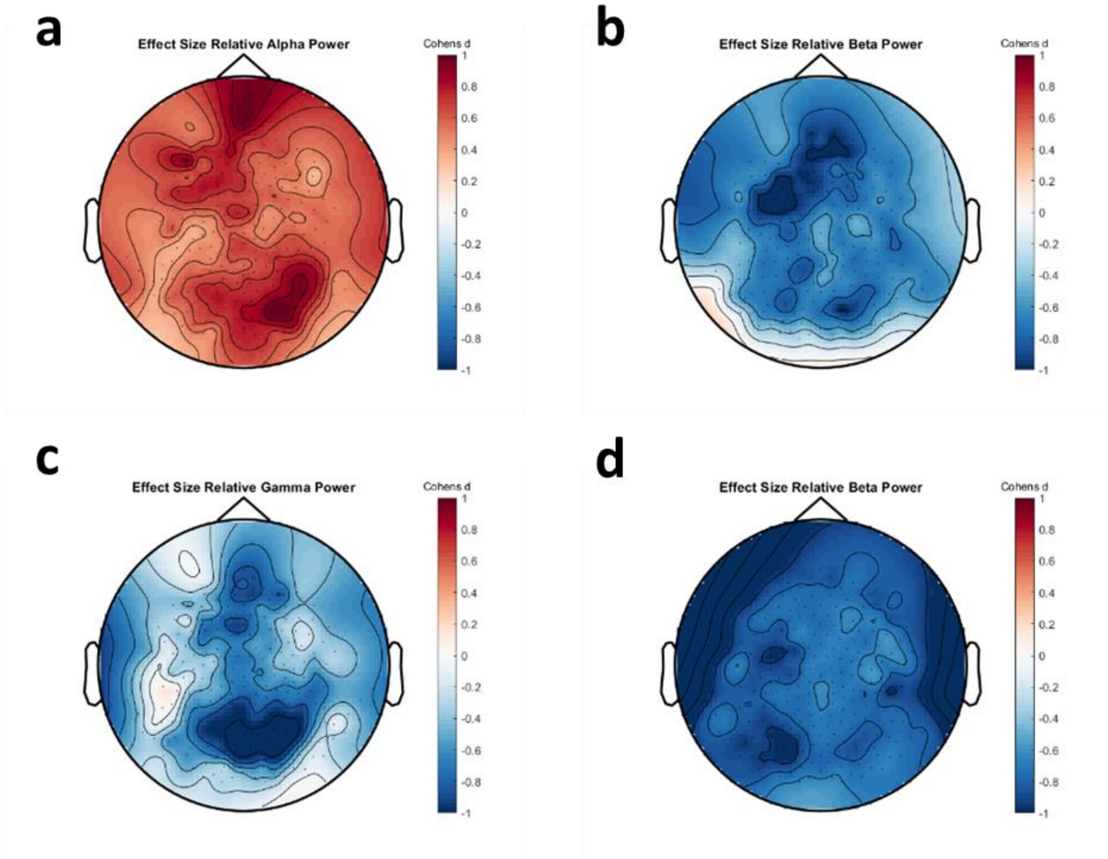

Supplementary Figure 1. Cohen's d maps for cluster correction in alpha, beta, gamma in eyes open condition and beta in eyes closed condition.

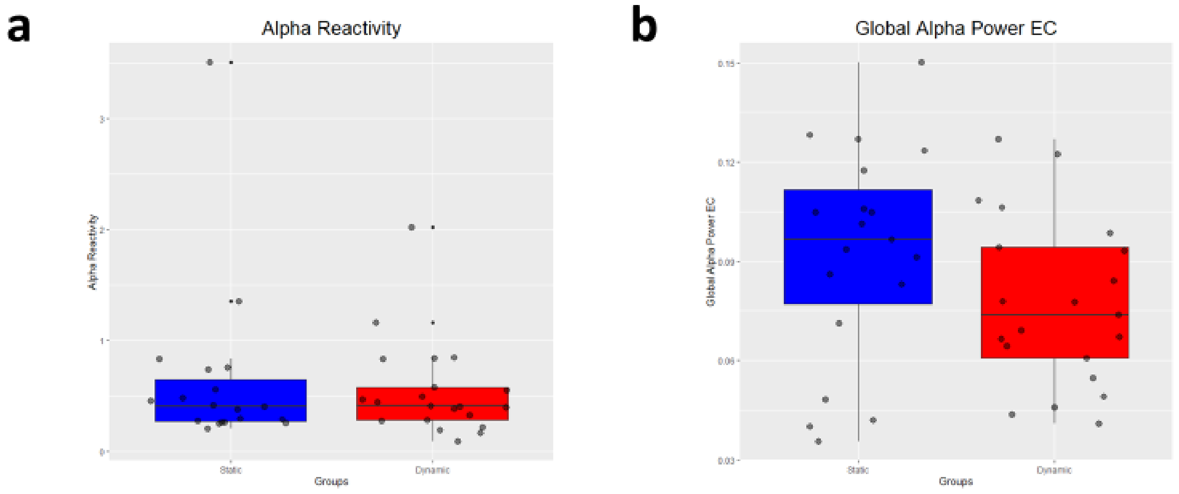

Supplementary Figure 2. (**a**) Alpha reactivity values by group (static n=19, median = 0.410, dynamic n=21, median = 0.403). N=40. (**b**) Global alpha power eyes closed by group (static n=19, median = 0.09, dynamic n=21, median = 0.07). N=40.

	Dynamic	Static	statistic	p-value
**Sex**				
F	16(76.2%)	9(47.4%)	X^2 = 2.41	0.12
M	5(23.8%)	10(52.6%)		
**Age**				
Mean(SD)	22.9(1.50)	25.3(2.75)	W = 69	**0,0004****
Median(min,max)	23(20, 25)	24(22, 33)		
Missing	1(4%)	0(0%)		
**Education(years)**				
Mean(SD)	16.1(1.30)	16.7(1.06)	W = 149	0.161
Median(min,max)	16(14, 18)	17(14, 18)		
**Coffee Units - Daily Mean (Zangrossi et al., 2021)**				
Mean(SD)	1.64(0.989)	1.51(1.14)	W = 206	0.630
Median(min,max)	2(0, 3.50)	1.50(0, 4)		
Missing	0(0%)	1(5.3%)		
**Coffee Before The Session (Zangrossi et al., 2021)**				
Yes	7(33%)	8(42.1%)	X^2 = 0.09	0.75
No	14(66.7%)	11(57.9%)		
**Alcool Units - Weekly Mean (Zangrossi et al., 2021)**				
Mean(SD)	2.54(2.99)	4.19(3.70)	W = 135	0.12
Median(min,max)	1(0, 7)	3(0, 14)		
Missing	0(0%)	1(5.3%)		
**Cigarettes - Daily Mean (Zangrossi et al., 2021)**				
Mean(SD)	2.42(4.41)	2.72(4.25)	W = 191.5	0.94
Median(min,max)	0(0, 15)	0(0, 10)		
Missing	0(0%)	1(5.3%)		
**Smoke Before The Session (Zangrossi et al., 2021)**				
Yes	17(81%)	12(63.2%)	X^2 = 0.81	0.36
No	4(19%)	7(36.8%)		
**DASS Anxiety Score (Zangrossi et al., 2021)**				
Mean(SD)	6.14(4.28)	5,17(3.09)	W = 202	0.72
Median(min,max)	5(1, 15)	4.50(0, 12)		
Missing	0(0%)	1(5.3%)		
**DASS Depression Score (Zangrossi et al., 2021)**				
Mean(SD)	8(5.04)	5.33(4.43)	W = 247	0.10
Median(min,max)	8(1, 20)	4.50(0, 13)		
Missing	0(0%)	1(5.3%)		
**DASS Stress Score (Zangrossi et al., 2021)**				
Mean(SD)	11(4.07)	8.22(4.45)	W = 262.5	0.03
Median(min,max)	12(2, 17)	7.50(3, 18)		
Missing	0(0%)	1(5.3%)		
**Sleep Hours - Night Before The Session (Current Study)**				
Mean(SD)	7.38(0.77)	7.26(1.05)	W = 206	0.86
Median(min,max)	7(6, 9)	7(5, 9)		
**Coffee Units - Daily Mean (Current Study)**				
Mean(SD)	1.43(1.09)	1.82(1.45)	W = 167	0.37
Median(min,max)	1.50(0, 4)	2(0, 6)		

**Alcool Units - Weekly Mean (Current Study)**<br>Mean(SD)<br>Median(min,max)	 2.20(2.60)<br>1.50(0, 7)	 3.21(2.76)<br>3(0, 7)	W = 158	0.25
**Cigarettes - Daily Mean (Current Study)**<br>Mean(SD)<br>Median(min,max)<br>Missing	 1.74(3.59)<br>0(0, 12)<br>0(0%)	 2.50(5.22)<br>0(0, 20)<br>1(5.3%)	W = 186	0.92

Supplementary Table 3. Demographics and personality information by group, along with statistics (Wilcoxon Rank sum test or chi-squared) and p-values resulting from group contrasts. P-values are uncorrected, with bold for significant differences after accounting for multiple comparisons with Benjamini and Hochberg's FDR ([1]). N= 40.

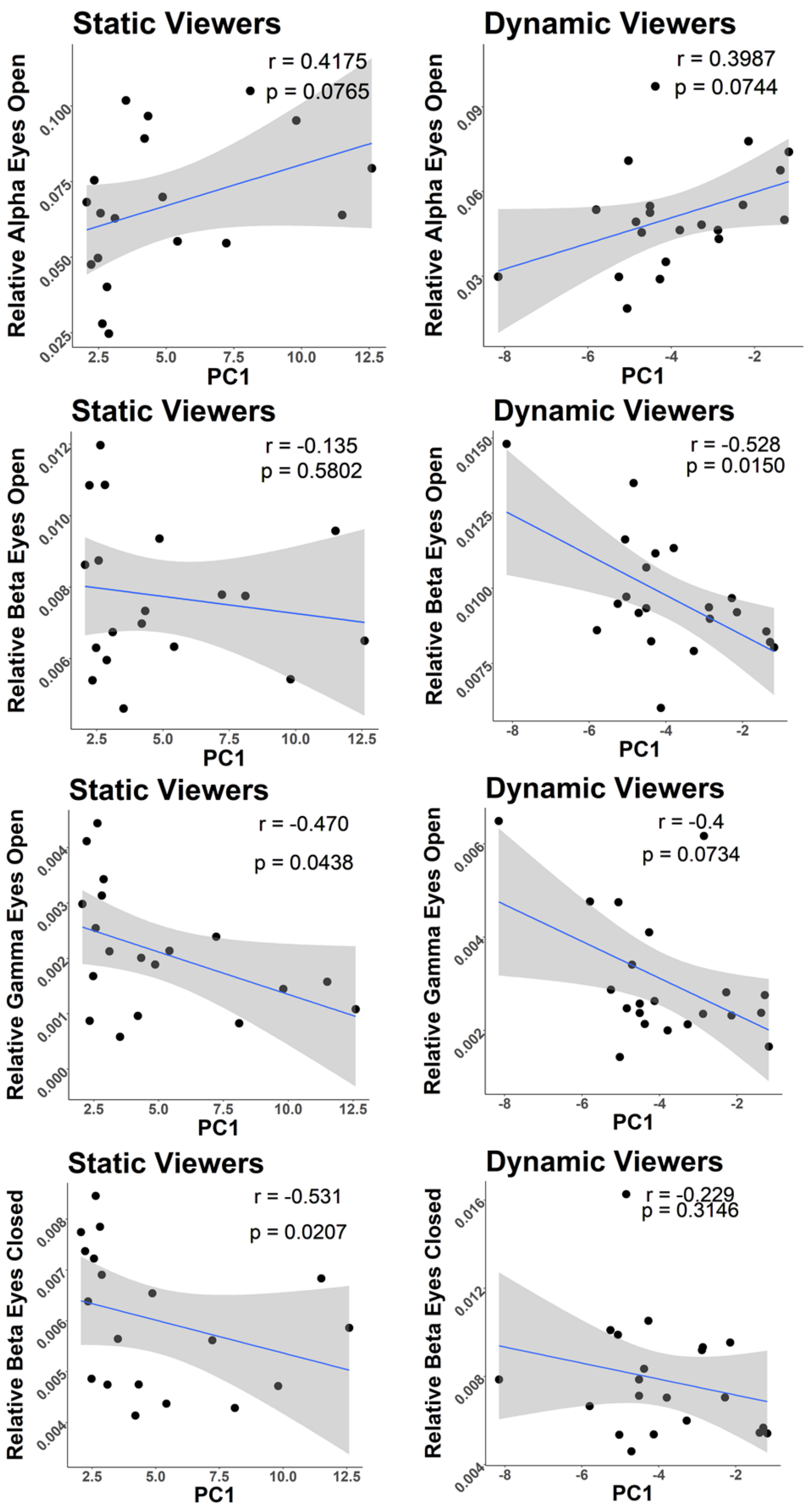

Supplementary Figure 3. Spearman's rank correlation between PC1 and frequency values by group (with Spearman's r, p-value and 95 % CI). N= 40.

Supplementary Methods

To assess LRTCs, we applied a Detrended Fluctuation Analysis on both EEG sensor data and eye-tracking data. Detrended Fluctuation Analysis is a technique to assess long-range temporal correlations in non-stationary signals ([2]). This technique extracts a power-law exponent, typically ranging between 0.5 and 1 in brain signals ([3]) and eye movements time-series ([4]). While an exponent of 0.5 index an uncorrelated signal (i.e., white noise), an exponent of 1 index strong long-range temporal correlations ([2,5]).

Pre-processed eye-tracking data were transformed into a time-series comparable to those extracted from the EEG signal (cfr.[6]).

For this step, we considered the fixed viewing time (2s) and excluded the rest of the exploration time per image. Images containing missing data for at least one subject are excluded, resulting in a final pool of 128 images and a total number of 31200 time points.

Fixations were extracted with the fixation detection algorithm implemented in R in the package 'saccades' (https://github.com/tmalsburg/saccades), in which the detection is obtained using a velocity-based algorithm for saccade detection proposed by Engbert and Kliegl ([7]). Anything between two saccades is considered a fixation.

A time-series was created, in which every time point is represented (120 frames per second). Timepoints in which a fixation was detected in the previous step were assigned a zero value, while all the other points, which represent eye movements, were assigned value one.

High-density EEG data received an additional pre-processing (cfr. [6]) before the DFA: first, the EEG signals were bandpass filtered in the frequency of interest (7.5-12 Hz, order 66), and then the amplitude envelope was extracted using a Hilbert transform. We choose the alpha band as the frequency of interest for this step because, based on the existing literature (e.g., [6]) this is the frequency band showing the strongest association with behavioural data, both at rest and during task.

To avoid spurious temporal correlations induced by the filter, the filter order and the lowest fitting window were chosen based on a simulation. We simulated 1000 white Gaussian noises with the same length of the signal and applied the filter. Filter order was chosen following Hardstone et al.[5] as two cycles of the lower bound of the frequency of interest (2 cycles of 7.5 Hz); while the lowest fitting window was chosen as the one where DFA exponents deviate from the expected known value (i.e., 0.5) in the white Gaussian noise simulation. The resulting lowest fitting window is 2.36 seconds (590 time points). For the 2.36-64 s intervals, the scaling exponents obtained for the white Gaussian noise simulation with a FIR pass-band filter (cut-off frequencies: 7.5-12, order: 66) had a mean value of 0.508 ±0.025. The expected DFA exponent for a white Gaussian noise is 0.50. For the high end of the fitting window, the maximum length allowed has to be at most N/4, where N is the total number of time points ([5]); this is because the number of segments in the averaging procedure would become otherwise too small and thus statistically unreliable. Therefore, the highest fitting window was determined as N/4 time points, as computed from the shortest signal (i.e. behavioural data), resulting in a maximum fitting window of 64 sec.

Next, both time-series are integrated (i.e., the mean-centred cumulative sum is computed):

(1)

$$x(k) = \sum_{t=1}^{k} a(t) - < a >$$

Where a(t) is the value of the time series at point t and <a> is the mean of the time series.

The signal is then split into 50 logarithmically spaced time-windows varying from 2.36 to 64 sec. Each segment of the integrated data is locally fitted to a linear function and the mean-squared residual is computed:

(2)

$$F(\Delta t) = \sqrt{\frac{1}{N}\sum_{i=1}^{N}[y(i) - y_{\Delta t}(i)]^2}$$

where N is the total number of data points. The scaling exponent is defined as the slope of the linear regression of the function in log-log coordinates, estimated using a least-squares algorithm.

Supplementary References

1. Benjamini, Y. & Hochberg, Y. Controlling the False Discovery Rate : A Practical and Powerful Approach to Multiple Testing Author (s): Yoav Benjamini and Yosef Hochberg. *J. R. Stat. Soc.* **57**, 289–300 (1995).
2. Peng, C. K. *et al.* Mosaic organization of DNA nucleotides. *Phys. Rev. E* **49**, 1685–1689 (1994).
3. Linkenkaer-hansen, K., Nikouline, V. V, Palva, J. M. & Ilmoniemi, R. J. Long-Range Temporal Correlations and Scaling Behavior in Human Brain Oscillations. **21**, 1370–1377 (2001).
4. Liang, J. *et al.* Scaling of horizontal and vertical fixational eye movements. 1–6 (2005) doi:10.1103/PhysRevE.71.031909.
5. Hardstone, R. *et al.* Detrended fluctuation analysis : a scale-free view on neuronal oscillations. **3**, 1–13 (2012).
6. Palva, J. M., Zhigalov, A., Hirvonen, J., Korhonen, O. & Linkenkaer-hansen, K. Neuronal long-range temporal correlations and avalanche dynamics are correlated with behavioral scaling laws. **110**, 3585–3590 (2013).
7. Engbert, R. & Kliegl, R. Microsaccades uncover the orientation of covert attention. **43**, 1035–1045 (2003).
