# Supplementary material for: One-year-later spontaneous EEG features predict visual exploratory human phenotypes
Source: Commun Biol. 2022 Dec 12;5:1361. doi: 10.1038/s42003-022-04294-9 (PMC9744741; doi:10.1038/s42003-022-04294-9)
Supplement: Supplementary file 2 — Reporting Summary [file 42003_2022_4294_MOESM2_ESM.pdf]

nature portfolio

Corresponding author(s): Maurizio Corbetta

Last updated by author(s): Nov 2, 2022

Reporting Summary

Nature Portfolio wishes to improve the reproducibility of the work that we publish. This form provides structure for consistency and transparency in reporting. For further information on Nature Portfolio policies, see our Editorial Policies and the Editorial Policy Checklist.

Statistics

For all statistical analyses, confirm that the following items are present in the figure legend, table legend, main text, or Methods section.

n/a	Confirmed	
☐	☒	The exact sample size (n) for each experimental group/condition, given as a discrete number and unit of measurement
☐	☒	A statement on whether measurements were taken from distinct samples or whether the same sample was measured repeatedly
☐	☒	The statistical test(s) used AND whether they are one- or two-sided<br>*Only common tests should be described solely by name; describe more complex techniques in the Methods section.*
☒	☐	A description of all covariates tested
☐	☒	A description of any assumptions or corrections, such as tests of normality and adjustment for multiple comparisons
☐	☒	A full description of the statistical parameters including central tendency (e.g. means) or other basic estimates (e.g. regression coefficient) AND variation (e.g. standard deviation) or associated estimates of uncertainty (e.g. confidence intervals)
☐	☒	For null hypothesis testing, the test statistic (e.g. F, t, r) with confidence intervals, effect sizes, degrees of freedom and P value noted<br>*Give P values as exact values whenever suitable.*
☒	☐	For Bayesian analysis, information on the choice of priors and Markov chain Monte Carlo settings
☒	☐	For hierarchical and complex designs, identification of the appropriate level for tests and full reporting of outcomes
☐	☒	Estimates of effect sizes (e.g. Cohen's d, Pearson's r), indicating how they were calculated

Our web collection on statistics for biologists contains articles on many of the points above.

Software and code

Policy information about availability of computer code

Data collection	NetStation Acquisition Software 4.3<br>For eye tracking data recorded in the previous experiment see reference 11
Data analysis	MATLAB (2018b), using functions from the following softwares : EEGLAB (version 14.1.2, reference 53), FieldTrip (reference 62)<br>R 4.1.0

For manuscripts utilizing custom algorithms or software that are central to the research but not yet described in published literature, software must be made available to editors and reviewers. We strongly encourage code deposition in a community repository (e.g. GitHub). See the Nature Portfolio guidelines for submitting code & software for further information.

Data

Policy information about availability of data

All manuscripts must include a data availability statement. This statement should provide the following information, where applicable:

- Accession codes, unique identifiers, or web links for publicly available datasets
- A description of any restrictions on data availability
- For clinical datasets or third party data, please ensure that the statement adheres to our policy

Data are available upon request from the corresponding author.

Field-specific reporting

Please select the one below that is the best fit for your research. If you are not sure, read the appropriate sections before making your selection.

☐ Life sciences ☒ Behavioural & social sciences ☐ Ecological, evolutionary & environmental sciences

For a reference copy of the document with all sections, see nature.com/documents/nr-reporting-summary-flat.pdf

Behavioural & social sciences study design

All studies must disclose on these points even when the disclosure is negative.

Study description	All data in this manuscript are quantitative, based on continous EEG recordings and eye-tracking data recorded in a previous experiment (reference 11).
Research sample	We recruited n=43 students of University of Padova (mean age 24.11 years, sd 2.41, 16 males) selected from a sample of n=120 analyzed in a previous study as those more representative of different viewing styles (see reference 11 for details and eligibility criteria). In the present study, three subjects were excluded for poor data quality, resulting in a final sample of 40 (19 static viewers and 21 dynamic viewers).
Sampling strategy	Sample size was not determined a priori. Since our aim was to compare EEG resting state dynamics between static and dynamic viewers, participants were chosen as those more representative of the two behavioural phenotypes emerged from the previous study (reference 11).
Data collection	Participants' data were collected using signal acquisition software (NetStation Acquisition 4.3). Participants sat in a sound-shielded Faraday cage and completed two consecutive recordings : eyes open (10 minutes) followed by eyes closed (10 minutes), followed by a memory task which is not discussed in the present study. The EEG was recorded with a Geodesic system (Hydrocel 256 channel) and an MR-compatible NetAmp GES 400.
Timing	Start Jan 16th 2019<br>End May 13th 2019
Data exclusions	Three subjects were excluded due to low quality of EEG recordings
Non-participation	No participant dropped out during the acquisition phase.
Randomization	Group allocation was determined in reference 11.

Reporting for specific materials, systems and methods

We require information from authors about some types of materials, experimental systems and methods used in many studies. Here, indicate whether each material, system or method listed is relevant to your study. If you are not sure if a list item applies to your research, read the appropriate section before selecting a response.

Materials & experimental systems

n/a	Involved in the study	
☒	☐	Antibodies
☒	☐	Eukaryotic cell lines
☒	☐	Palaeontology and archaeology
☒	☐	Animals and other organisms
☐	☒	Human research participants
☒	☐	Clinical data
☒	☐	Dual use research of concern

Methods

n/a	Involved in the study	
☒	☐	ChIP-seq
☒	☐	Flow cytometry
☒	☐	MRI-based neuroimaging

Human research participants

Policy information about studies involving human research participants

Population characteristics	See above
Recruitment	Participants were recruited from a previous sample of n=120 (reference 11), as those more representative of different viewing styles.
Ethics oversight	Department of General Psychology (DPG), University of Padova

Note that full information on the approval of the study protocol must also be provided in the manuscript.
